# *Delta* and *jagged* are candidate target genes of RNAi biopesticides for the control of *Nilaparvata lugens*

**DOI:** 10.3389/fbioe.2022.1023729

**Published:** 2022-11-18

**Authors:** Xifa Yang, Shaokai Liu, Wenhui Lu, Mengfang Du, Zhuangzhuang Qiao, Zhen Liang, Yiting An, Jing Gao, Xiang Li

**Affiliations:** ^1^ State Key Laboratory of Wheat and Maize Crop Science/Henan International Laboratory for Green Pest Control/College of Plant Protection, Henan Agricultural University, Zhengzhou, China; ^2^ Key Laboratory of Integrated Pest Management on Crops in Northwestern Loess Plateau, Ministry of Agriculture/College of Plant Protection, Northwest A&F University, Yangling, China

**Keywords:** *Nilaparvata lugens*, RNAi biopesticide, Notch signaling pathway, *delta*, *jagged*

## Abstract

The brown planthopper (BPH; *Nilaparvata lugens*) is an important pest in rice cultivation, and chemical pesticide over-use and ineffectiveness of existing Bt transgenic rice against piercing-sucking insects make novel control methods necessary. RNA interference (RNAi) biopesticide is a new type of product with high efficiency and specificity and are simple to use. The Notch signaling pathway has extensive and important physiological functions and plays a key role in the development of insects. In this study, two key ligand genes of the Notch signaling pathway, *delta* (*dl*) and *jagged* (*jag*), were selected and their lethal effects and functional analysis were systematically evaluated using a stable short-winged population (Brachypterous strain) and a long-winged population (Macropterous strain) of BPHs. The full-length coding sequences of *Nldl* and *Nljag* comprised 1,863 and 3,837 base pairs, encoding 620 and 1,278 amino acids, respectively. The nucleic acid sequences of *Nldl* and *Nljag* were identical between the two strains. The expression levels of *Nldl* and *Nljag* were relatively high in the head of the nymphs, followed by those in the abdomen. Through RNAi treatment, we found that injection of BPH nymphs of both strains with ds*Nldl* (10–50 ng/nymph) or ds*Nljag* (100 ng/nymph) produced lethal or teratogenic effects. dsRNA treatment showed excellent inhibitory effects on the expression of target genes on days 1 and 5, suggesting that RNAi rapidly exhibits effects which persist for long periods of time in BPHs. Taken together, our results confirm the potential of *Nldl* and *Nljag* as target genes of RNAi biopesticides, and we propose optimized dosages for the control of BPHs.

## Introduction

The brown planthopper (BPH; *Nilaparvata lugens*, Hemiptera: Delphacidae) is a notorious pest in rice cultivation which can cause devastating damage. Adults and nymphs prefer to gather at the base of the rice plexus and suck the sap from stems and leaves, resulting in the loss of rice nutrients and decreased yield and quality (Alagar et al., 2007; Vanitha et al., 2011). In addition to direct feeding, BPHs are also a vector of pathogens including *rice grassy stunt virus*, *rice ragged stunt virus*, and *rice wilted stunt virus* (Hibino, 1995; Li et al., 2011). Honeydew excreted by BPHs contains large amounts of sugar, which is highly conducive to the propagation of bacteria and fungi, leading to mildew and wilting of rice (Sōgawa, 2003; Wei et al., 2010). Depending on habitat quality, adult BPHs can develop into a highly mobile long-winged morph or a highly fecund short-winged morph (Liu et al., 2020; Li et al., 2021). The differential formation of two wing morphs aggravates the damage caused by BPH and increases the difficulty of controlling its spread. In normal years, BPH infestation can cause a reduction of 10%–20% in rice production, and in severe cases, it can result in crop failure (Heong and Hardy, 2009).

RNA interference (RNAi) is a conserved regulatory mechanism mediated by RNA in eukaryotes. RNAi effectors target genes paired with homologous sequences and effectively suppress gene expression at the transcriptional or post-transcriptional level (Carthew and Sontheimer, 2009; Chen et al., 2010). RNAi biopesticide is a new type of product with high efficiency and specificity and which are easy to use, thus they are expected to constitute a scientific and technological revolution and facilitate sustainable agricultural development (Li et al., 2022). RNAi biopesticides can specifically interfere with the expression of designated genes of agricultural pests, pathogens, and weeds, thereby hindering their growth or preventing them from exerting harmful effects. In 2002, the feasibility of RNAi technology for gene interference in insects was first demonstrated using *Hemolin*-RNAi in *Hyalophora cecropia* (Bettencourt et al., 2002). Since then, numerous studies have demonstrated that exogenous synthesized dsRNA applied through feeding, injecting, or spraying can induce RNAi effects in various insects (Kim et al., 2015; Vogel et al., 2019).

Genes that are essential for insect growth and development can serve as targets for RNAi biopesticides. A very wide range of targets available for RNAi biopesticides can decrease the upfront development cost, which constitutes an important advantage of RNAi-based pesticides, compared to chemical pesticides. In a previous study, 290 insect genes were selected and silenced, and most of them led to a lethal effect (some could also cause larval maldevelopment), thus they may serve as insecticidal targets (Baum et al., 2007). Similarly, 100 potential target genes were isolated from random genes of *Tribolium castaneum*, and dsRNA injection inhibited the expression of these genes at the larval or pupal stage and resulted in >90% mortality (Ulrich et al., 2015).

The Notch protein is a heterodimer formed by a non-covalent bond between an extracellular domain subunit and an intracellular domain subunit (Kopan and Ilagan, 2009). Notch is a highly conserved transmembrane protein of vertebrates and invertebrates that can transmit signals unidirectionally and regulate cell proliferation, differentiation, metabolism, and apoptosis through collateral inhibition (Palomero et al., 2006; Lewis et al., 2007; Joshi et al., 2009). During insect ontogeny, Notch can affect normal development of the embryonic ectoderm, mesoderm, central nervous system, germ cells, eyes, wings, etc. (Bray, 2006; Irles et al., 2016; Hu et al., 2022). If Notch is abnormally expressed in the early stage of embryonic development, it may lead to abnormal embryonic differentiation and death (Kidd and Lieber, 2016).

Notch ligands are transmembrane proteins located on the surface of cells in close proximity to the Notch receptors, and they are also composed of extracellular and intracellular domains (Schroeter et al., 1998). When performing its function, Notch must bind to a ligand protein, i.e., Delta (Dl) or Jagged (Jag), and induce a conformational change to release the intracellular subunit. The modified intracellular subunits are transported into the nucleus and act as transcription factors to regulate the expression of downstream genes (Struhl and Adachi, 1998; Mumm et al., 2000; Mumm and Kopan, 2000; Gordon et al., 2007). Notch has both *cis-* and *trans*- regulation modes on its ligands. In addition, Notch exerts *cis*-inhibition effects on Jag, suggesting complex and diverse functions of Notch and emphasizing the critical role of ligands in signal transduction through the Notch signaling pathway (Matsuda and Chitnis, 2009; Fiuza et al., 2010; Yamamoto et al., 2012; Fleming et al., 2013).

In the current study, the cDNA sequences of the Notch ligand genes *Nldl* and *Nljag* were cloned and analyzed. Furthermore, the nucleotide homology and phylogenetic relationships of *Nldl/Nljag* between BPH and several common agricultural insects were investigated. The spatiotemporal expression profiles of *Nldl* and *Nljag* in BPH nymphs were measured by qPCR, and the effects of RNAi-*Nldl/Nljag* on the development and lethality of BPH were investigated by microinjection method. Taken together, this study evaluated the potential application of *Nldl* and *Nljag* as an RNAi biopesticides for BPH control.

## Materials and methods

### Insects

BPHs were collected from paddy fields of the Huazhong Agricultural University, Wuhan, China. According to Morooka and Tojo (1992), BPHs were reared in growth chambers containing rice seedlings (variety: Taichung Native 1) and were screened for more than seventy successive generations to cultivate and obtain a stable long-winged population (Macropterous strain, long-winged rate approximately 85% ± 5%) and a short-winged strain (Brachypterous strain, short-winged rate approximately 100%). The rearing conditions included a photoperiod of 14 h, 27 ± 1°C, and 60% ± 10% relative humidity. The BPHs of the Brachypterous and Macropterous strain tested in this study were generously donated by Professor Hongxia Hua from Huazhong Agricultural University.

### cDNA cloning and sequence analysis

Total RNA was isolated and extracted from a mixture of BPH adults and nymphs at different instar stages of the Brachypterous or Macropterous strain using TsingZol Reagent (Tsingke Biotech, Beijing, China). cDNA synthesis reactions were performed using 1 μg total RNA and an All-in-one First Strand cDNA Synthesis Kit Ⅲ for qPCR (Sevenbio, Beijing, China), according to the manufacturer’s instructions. High-accuracy sequences were amplified with 2 × KOD PCR MasterMix (Bioman, Beijing, China). The correctness of PCR products was confirmed by sequencing of Tsingke Biotech. The experimental reaction parameters refer to the default parameters given by the kits. Nucleic acid homology analysis was performed in the Blast module (https://blast.ncbi.nlm.nih.gov/Blast.cgi) of the National Center for Biotechnology Information (NCBI) database. Expasy (http://web.expasy.org) was employed to predict the sequence structures of *Nldl* and *Nljag*. MEGA 11.0 software was used to perform the sequence alignment and phylogenetic analysis with 1,000 replicates of the bootstrap test. STRING (https://cn.string-db.org/) was used to identify physical interactions and co-expression of the test proteins.

### qRT-PCR

BPH nymphs were divided into three body segments, i.e., the head, thorax, and abdomen, and additional appendages: the legs. qPCR was employed to investigate the expression profiles of *Nldl* and *Nljag* in these tissues of second- to fifth-instar BPH nymphs. Each sample contained 50 corresponding nymph tissues of the Macropterous strain or the Brachypterous strain. Total RNA was isolated and extracted from different tissues of BPHs using TRIzol reagent (Sangon Biotech, Shanghai, China). cDNA synthesis was performed using 1 μg total RNA and a TransScript Uni All-in-One First-Strand cDNA Synthesis SuperMix for qPCR (TransGen Biotech, Beijing, China), according to the manufacturer’s instructions. The amplification efficiencies of the qPCR primers of *Nldl* and *Nljag* were determined by using more than five concentrations of cDNA templates for amplification on QuantStudio3 Real-time PCR system. Detection of gene expression levels by qPCR was performed on an Applied Biosystems 7,500 Fast Real-time PCR system using the Hieff qPCR SYBR Green Master Mix (Yeasen Biotech, Shanghai, China) according to the manufacturer’s instructions. The thermal cycle conditions for qPCR were 95°C for 5 min, followed by 40 cycles of 95°C for 10 s and 60°C for 30 s. The experimental reaction parameters refer to the default parameters given by the kits. The housekeeping gene *Nlactin1* (GenBank accession EU179846.1) was used as a reference. The relative expression level of the test gene was calculated and compared according to Livak and Schmittgen (2001). Signal intensities of the target genes are presented as average values. Each sample was examined using three independent replicates, and each treatment was performed using three biological replicates. qPCR primers are listed in Table 1.

**TABLE 1:** Primers used in the current study.

Gene name (Genbank ID)	Primer sequence (5′→3′)	Product size
qPCR primers		
*Nlactin* (EU179846.1)	F: CCA​ACC​GTG​AGA​AGA​TGA​CC	296 bp
	R: GAT​GTC​ACG​CAC​GAT​TTC​AC	
*Nldelta* (KP196804)	F: CTC​CCA​CTC​CTA​CAC​CCA​ACA​CAA	91 bp
	R: TGC​TTC​TGA​GAC​CTG​CTC​CTG​T	
*Nljagged* (KP196803)	F: CGT​CCT​ACT​CTG​GCA​TCA​TCC​T	130 bp
	R: GTC​TGG​CAA​GTG​GCG​TTG​TAG​T	
dsRNA primers		
ds*Nldelta* (KP196804)	F: T7 + ATG​CCT​TCC​TGG​GTG​TGA​TGA	537 bp
	R: T7 + GCC​GTG​TTC​GGT​GTC​TTC​GCA	
ds*Nljagged* (KP196803)	F: T7 + GACGAATGTGAGCCATAC	466 bp
	R: T7 + CAA​GAG​CAA​CTG​TAA​CCA​T	
ds*GFP* (U76561)	F: T7 + GTA​AAC​GGC​CAC​AAG​TTC​AG	451 bp
	R: T7 + TCG​GCC​ATG​ATA​TAG​ACG​TT	

T7 sequence: GGA​TCC​TAA​TAC​GAC​TCA​CTA​TAG​G.

### dsRNA synthesis and microinjection

The PCR template (cDNA) for *Nldl* and *Nljag* was amplified using gene-specific primers containing T7 polymerase sites and with 2 × Taq Plus PCR Master Mix (Solarbio, Beijing, China). dsRNA was generated using a T7 RNAi Transcription Kit (Vazyme Biotech, Nanjing, China) and was dissolved in diethyl pyrocarbonate-treated. Third-instar nymphs were injected with ds*Nldl* (10, 25, or 50 ng/nymph) or ds*Nljag* (100 ng/nymph) dissolved in 20 nl volume of the nuclease-free water on the second day after ecdysis from the ventral side of the thorax using a Nanoliter 2010 microinjector (WPI, Sarasota, United States). An equivalent amount of ds*GFP* (GenBank accession U76561) was injected as a control. The survival numbers of injected BPHs were recorded daily until adult emergence. Three replicates were used per treatment, and each replicate comprised 50 nymphs. The whole bodies of five treated-BPH were sampled randomly on the first and fifth day after injection (corresponding to third- and fifth-instar nymphs) for evaluating RNAi delivery efficiency by qPCR. Adult phenotypes were observed and photographed using a stereomicroscope (Olympus szx16, Tokyo, Japan). Primers used for dsRNA synthesis are shown in Table 1.

### Statistical analyses

Statistical analyses were performed using SPSS 20.0 software (IBM, Armonk, United States). Differences between treatments regarding RNAi efficiency and survival responses were compared using Student’s *t*-tests. Expression profiles of *Nldl* or *Nljag* in the same tissue of BPH nymph at different instars were analyzed using one-way analysis of variance followed by Tukey’s test for multiple comparisons. Statistical significance is reported at *p* < 0.05.

## Results

### Sequence cloning and analysis of *Nldl* and *Nljag*

**FIGURE 1:**
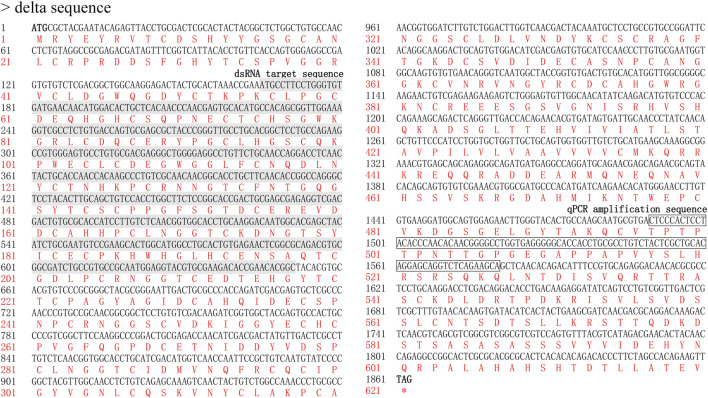
Nucleotide and deduced amino acid sequence of *Nldelta*. The target sequence of ds*Nldelta* is indicated in grey; the fragment amplified by qPCR is indicated by a box.

**FIGURE 2:**
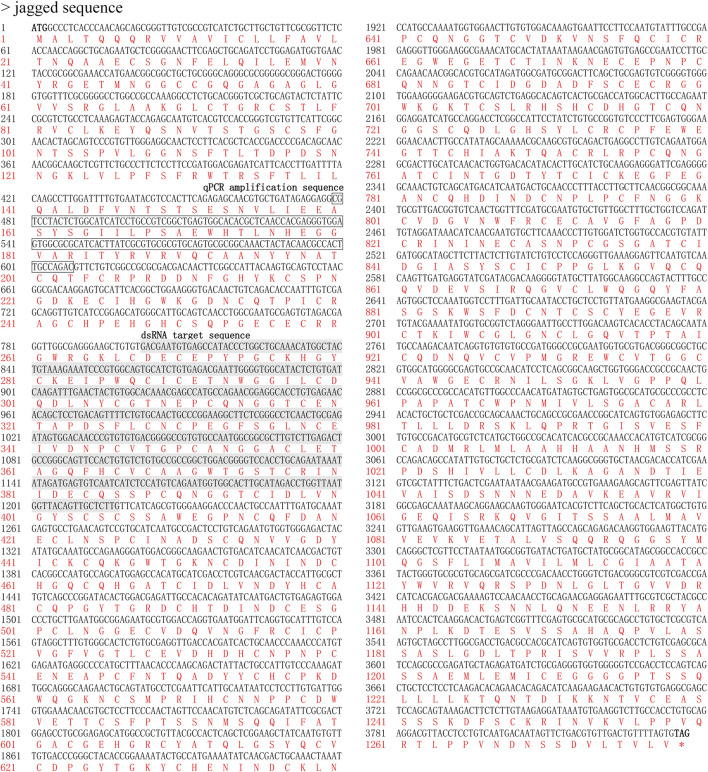
Nucleotide and deduced amino acid sequences of *Nljagged*. The target sequence of ds*Nljagged* is indicated in grey; the fragment amplified by qPCR is indicated by a box.

cDNA of *dl* and *jag* was amplified using BPHs of the Brachypterous and Macropterous strain, respectively. The full-length coding sequence of *Nldl* comprised 1,863 base pairs (bp), encoding 620 amino acids, with a molecular weight of 67.25 kDa (Figure 1). The *Nljag* full-length coding sequence comprised 3,837 bp, encoding 1,278 amino acids; its molecular weight was 137.83 kDa (Figure 2). The nucleic acid sequences of *Nldl* and *Nljag* were identical between the two strains.

### Phylogenetic analysis of *delta* and *jagged*

**FIGURE 3:**
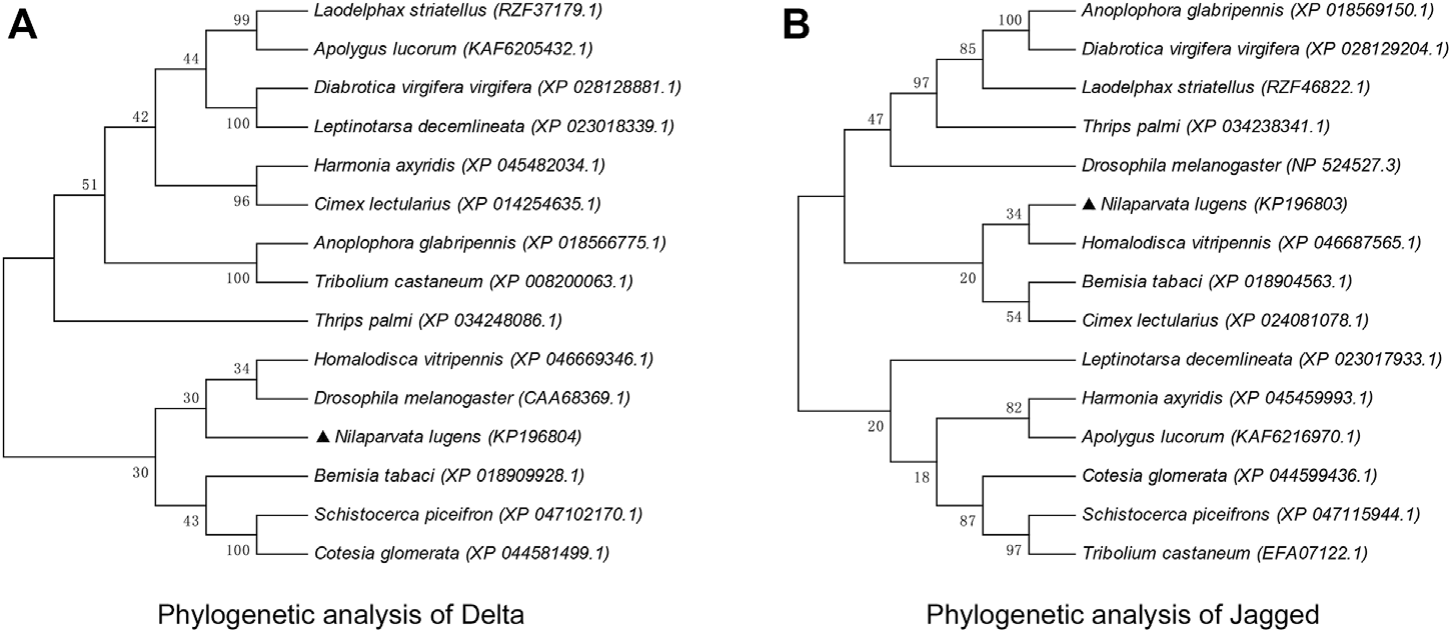
Phylogenetic analysis of Delta **(A)** and Jagged **(B)**. The phylogenetic tree was constructed through neighbor-joining using MEGA11.0 software. The percentage of replicate trees in which the associated taxa clustered together in the bootstrap test (1,000 replicates) is shown above the branches. Amino acid sequences of Dl and Jag (and its homologous protein Ser) were retrieved from the NCBI database; the respective GenBank accession numbers are shown in brackets after the Latin species name.

The amino acid sequences of Dl and Jag of 15 agriculturally important insects (including natural enemies and pests) of the Hemiptera, Coleoptera, Diptera, Orthoptera, Hymenoptera, etc. were collected and used for phylogenetic analyses. The evolutionary pattern of NlDl and NlJag did not produce a consistent picture. With regard to Dl, *N. lugens* appeared to be more closely related to *Drosophila melanogaster* and *Homalodisca vitripennis* (Figure 3A). However, the Jag sequence of *N. lugens* suggested that it was more closely related to Hemipteran insects such as *H. vitripennis*, *Bemisia tabaci*, and *Cimex lectularius* (Figure 3B). Through the nucleic acid homology analysis in the Blast module of the NCBI database, it was found that the nucleotide sequence of *dl* had no more than 76% homology with other species (mainly insects), while the nucleotide sequence homology of *jag* and other species (mainly insects) is no more than 71%.

### Expression analysis of *Nldl* and *Nljag* in brown planthopper

**FIGURE 4:**
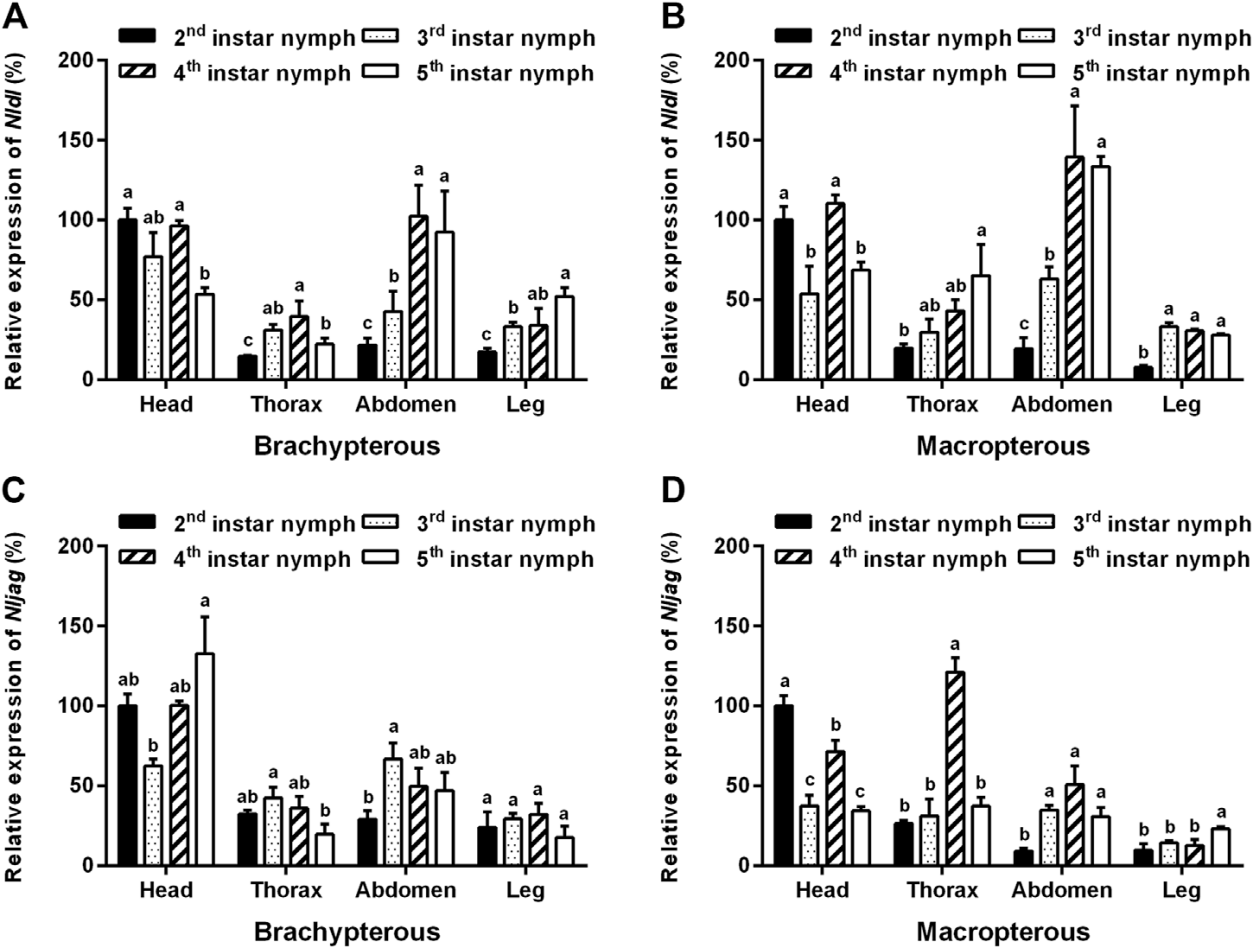
Spatiotemporal expression analysis of *Nldelta* and *Nljagged* in BPH nymphs by qPCR detection. **(A)** Expression profile of *Nldelta* in the Brachypterous strain. **(B)** Expression profile of *Nldelta* in the Macropterous strain. **(C)** Expression profile of *Nljagged* in the Brachypterous strain. **(D)** Expression profile of *Nljagged* in the Macropterous strain. Shown are the means ± standard error of three biological replicates, with 50 respective nymph tissues per replicate. *Nlactin1* was used as a housekeeping gene for quantitation of relative expression levels of the test genes. Data represent the mean ± standard error (*n* = 3). The expression level of the test gene in the head of the second-instar nymph was defined as 100%. Different letters on the top of the columns indicate significant differences in expression levels between instars in the same tissue, as assessed using Tukey’s test (*p* < 0.05).

The amplification specificity and amplification efficiency of the qPCR primers of *Nldl* and *Nljag* were first examined. The results showed that the amplification efficiencies of the primers of *Nldl* and *Nljag* were 90.65% and 91.44%, respectively, and no non-specific amplification occurred, confirming the availability of these two pairs of qPCR primers. The expression levels of *Nldl* and *Nljag* were relatively high in the head, followed by those in the abdomen (Figure 4). *Nldl* expression was relatively high in the abdomen of fourth- to fifth-instar nymphs (Figures 4A,B). The expression of *Nljag* in the thorax of fourth-instar nymphs of the Macropterous strain was significantly higher than that in other instars (Figure 4D).

### RNAi of *Nldl* and *Nljag* in brown planthopper

**FIGURE 5:**
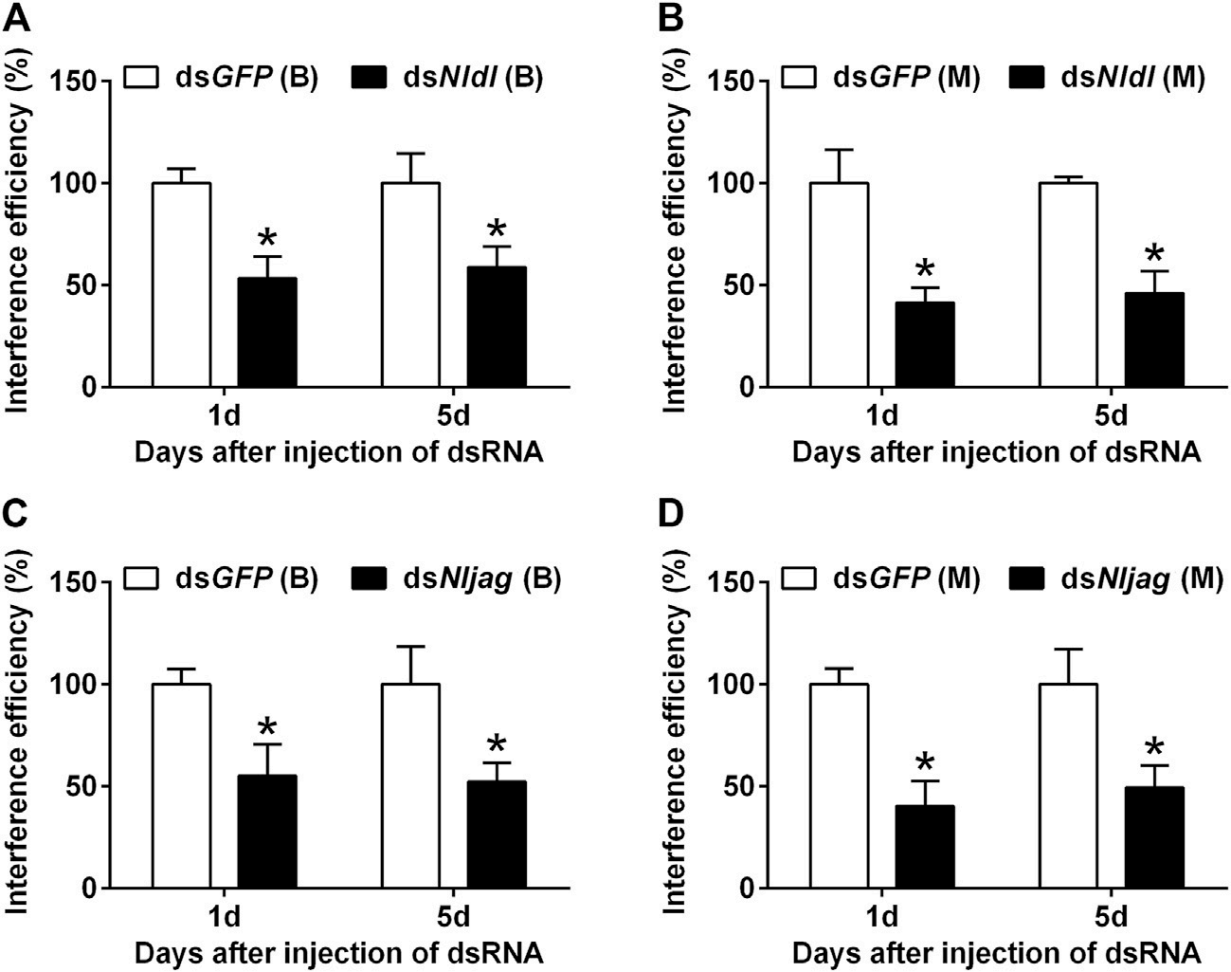
Interference efficiency of *Nldelta*-RNAi and *Nljagged*-RNAi in BPH nymphs. **(A)** Interference efficiency of *Nldelta* in the Brachypterous strain. **(B)** Interference efficiency of *Nldelta* in the Macropterous strain. **(C)** Interference efficiency of *Nljagged* in the Brachypterous strain. **(D)** Interference efficiency of *Nljagged* in the Macropterous strain. Shown are the means ± standard error of three replicates, with ten nymphs per replicate. Third-instar nymphs were injected with 25 ng ds*Nldelta* or 100 ng ds*Nljagged*. Equivalent ds*GFP* was used as a control. *Nlactin1* was used as a housekeeping gene quantitation of relative expression levels of the test genes. Data represent the mean ± standard error (*n* = 3). Asterisks indicate significant differences in expression levels between ds*GFP* and ds*Nldelta*/ds*Nljagged*, as assessed using Student’s *t*-tests (*p* < 0.05).

Different doses of dsRNA were injected from the ventral side of thorax into the third-instar BPH nymphs of the Brachypterous and Macropterous strain, respectively. dsRNA delivery efficiency was measured on the first and fifth day after treatment. *Nldl*-RNAi and *Nljag*-RNAi showed excellent interference efficiency and persistent effects (Figure 5). The expression levels of *Nldl* and *Nljag* decreased by 46.65% and 44.48%, respectively, in the Brachypterous strain on day 1 after treatment, and they decreased by 58.67% and 59.79% in the Macropterous strain (Figures 5A,C). On day 5, the expression levels of *Nldl* and *Nljag* in the Brachypterous strain were 58.67% and 52.35% of those in the control group, and 46.04% and 49.37% in the Macropterous strain, respectively (Figures 5B,D).

**FIGURE 6:**
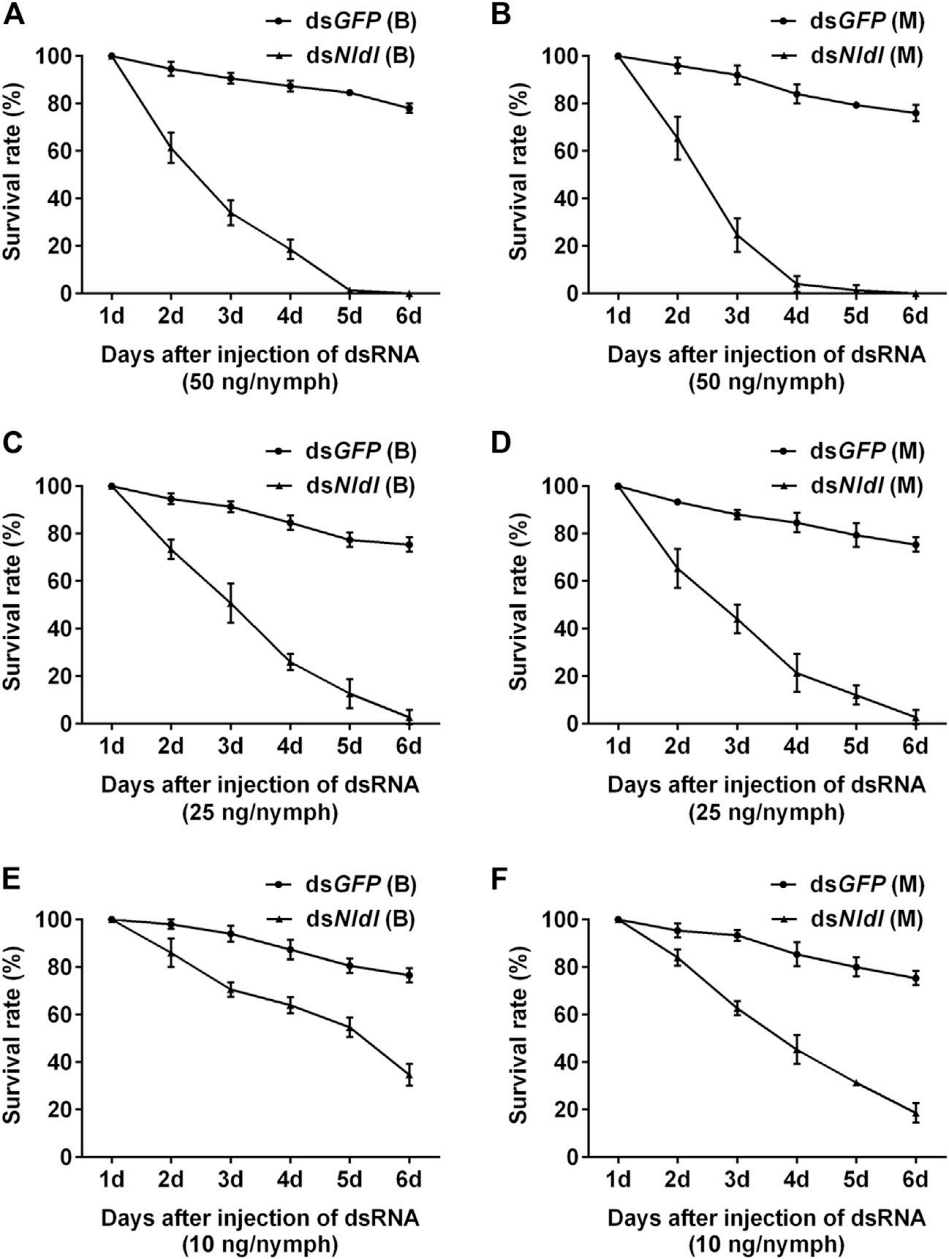
Survival rates of BPH developed from nymphs treated with ds*Nldelta*. **(A,B)**: Survival rate of 50 ng ds*Nldelta* injection of the Brachypterous **(A)** and Macropterous strain **(B)**. **(C,D)**: Survival rate of 25 ng ds*Nldelta* injection of the Brachypterous **(C)** and Macropterous strain **(D)**. **(E,F)**: Survival rate of 10 ng ds*Nldelta* injection of the Brachypterous **(E)** and Macropterous strain **(F)**. The nymphs injected with dsRNA were third-instar nymphs on the second day after ecdysis. The test nymphs began transforming to adults on the sixth day, and all surviving nymphs developed into adults on the seventh day. We therefore recorded nymph survival rates on the sixth day after treatment and additionally recorded the emergence rates. Equivalent ds*GFP* was used as a control. Each value is a mean of three replicates, with 50 nymphs per replicate. Data represent the mean ± standard error (*n* = 3).

**FIGURE 7:**
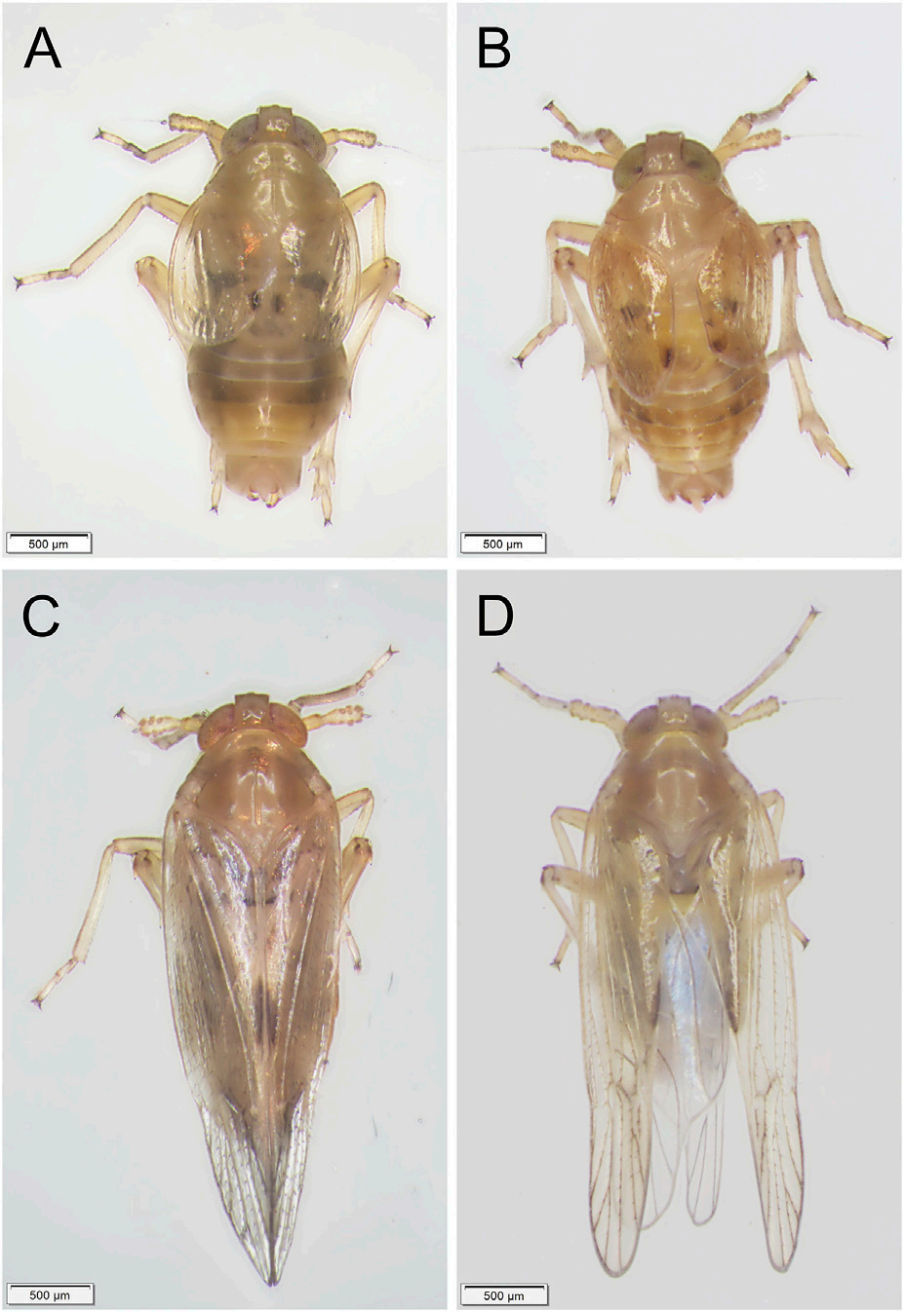
Phenotypes of BPH adults developed from nymphs treated with ds*Nldelta*. **(A,B)**: Phenotypes of BPH adults of the Brachypterous strain with ds*GFP*-treatment **(A)** or ds*Nldelta*-treatment **(B)**. **(C,D)**: Phenotypes of BPH adults of the Macropterous strain with ds*GFP*-treatment **(C)** or ds*Nldelta*-treatment **(D)**. Third-instar nymphs were injected with 25 ng ds*Nldelta*. Equivalent ds*GFP* was used as a control.

Third-instar nymphs treated with 50 ng ds*Nldl* produced very high mortality rates in the current study, and no nymphs reached the adult stage (Figures 6A,B). We thus reduced the injection dose to 25 ng/nymph, and approximately 2% of the Brachypterous and Macropterous strain nymphs successfully emerged (Figures 6C,D). These few survivors of both the long-winged and short-winged morphs produced a phenotype unable to close its wings (Figure 7). At 10 ng/nymph, the survival rate 6 days after treatment was 34.67% in the Brachypterous strain and 18.67% in the Macropterous strain, and 19.33% and 6.67% of the treated nymphs, respectively, successfully emerged and showed deformities rendering them unable to close their wings (Figures 6E,F). This constitutes a reference dosage for the application of ds*Nldl* in the field control of BPH.

**FIGURE 8:**
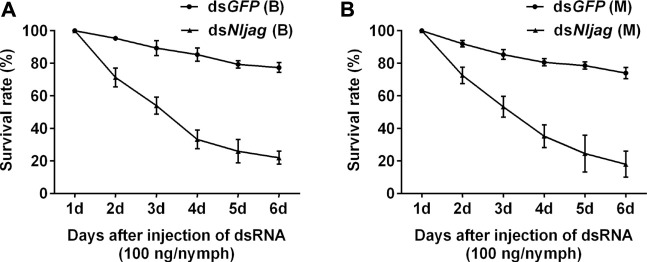
Survival rates of BPH developed from nymphs treated with ds*Nljagged*. **(A)**: Survival rate of 100 ng ds*Nljagged* injection of the Brachypterous strain. **(B)**: Survival rate of 100 ng ds*Nljagged* injection of the Macropterous strain. Third-instar nymphs on the second day after ecdysis were injected with dsRNA. Equivalent ds*GFP* was used as a control. Each value is a mean of three replicates, with 50 nymphs per replicate. Data represent the mean ± standard error (*n* = 3).

**FIGURE 9:**
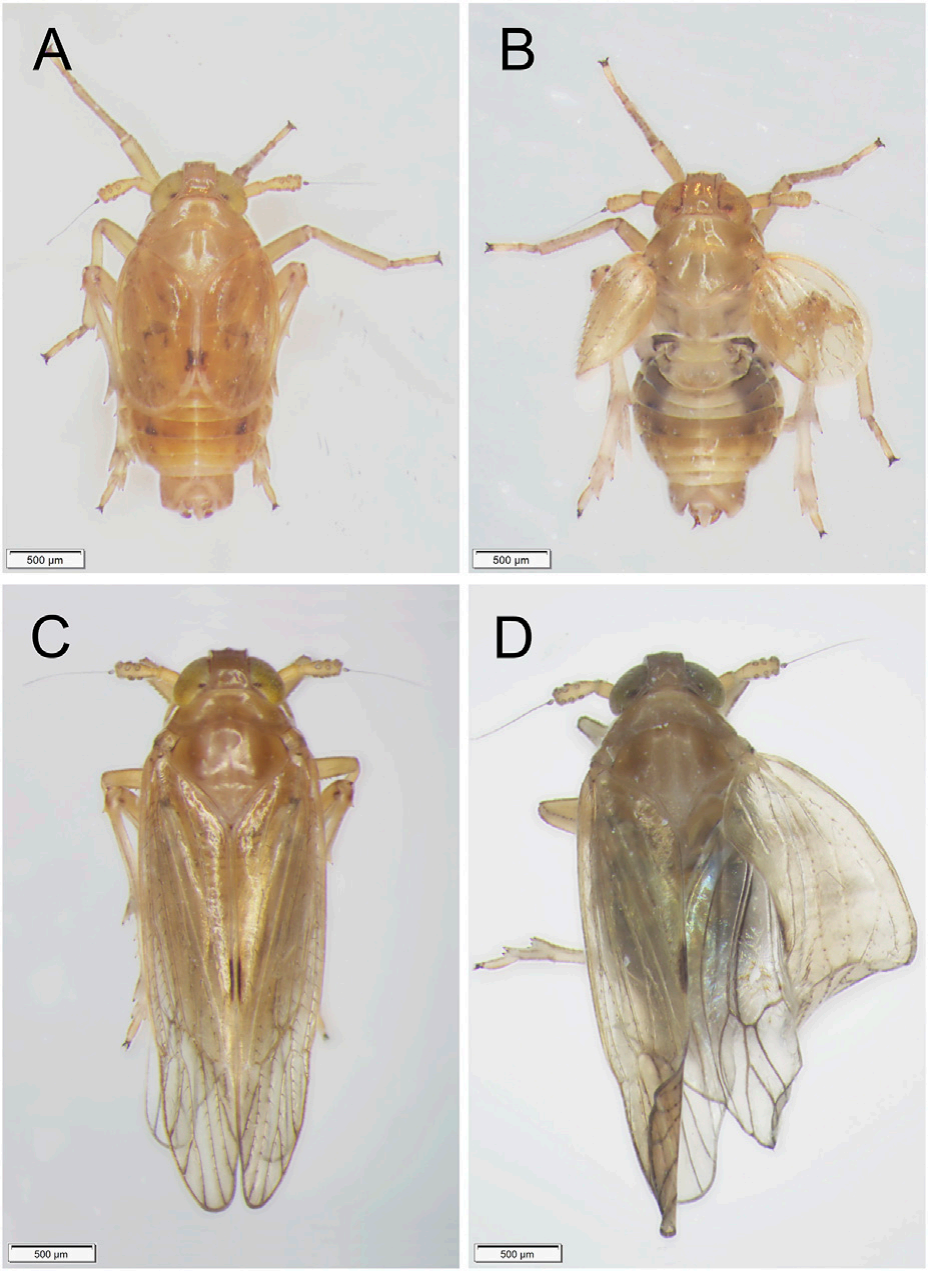
Phenotypes of BPH adults developed from nymphs treated with ds*Nljagged*. **(A,B)**: Phenotypes of BPH adults of the Brachypterous strain with ds*GFP*-treatment **(A)** or ds*Nljagged*-treatment **(B)**. **(C,D)**: Phenotypes of BPH adults of the Macropterous strain with ds*GFP*-treatment **(C)** or ds*Nljagged*-treatment **(D)**. Third-instar nymphs were injected with 100 ng ds*Nljagged*. Equivalent ds*GFP* was used as a control.

Injection with 100 ng ds*Nljag* caused a significant lethal effect on BPH nymphs. The survival rates after *Nljag-*RNAi injection in the Brachypterous and Macropterous strains were 22.00% and 18.00%, respectively, on day 6 after treatment, which were significantly lower than those of the control group (77.33%, *p* = 0.005328 and 74.00%, *p* = 0.002963, respectively) (Figure 8). On days 6 or 7 after ds*Nljag*-treatment, the surviving nymphs developed into adults, and all of them showed curled wings (Figure 9). Neither ds*Nldl* nor ds*Nljag* injection caused obvious developmental delay.

### Co-expressed protein prediction of NlDl and NlJag

**FIGURE 10:**
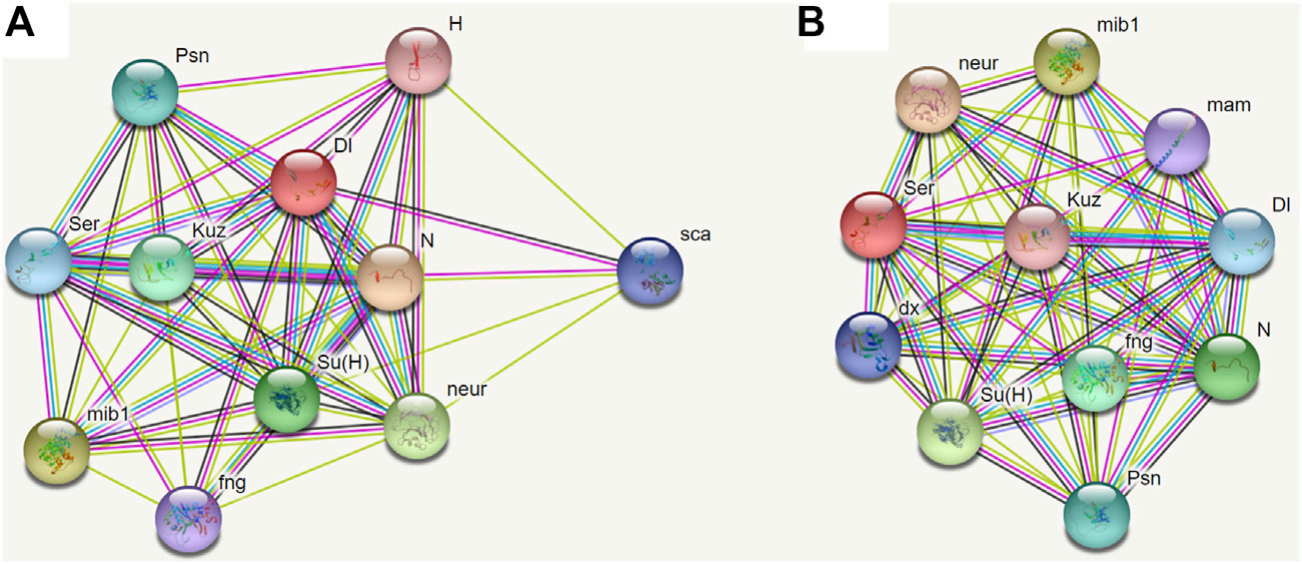
Prediction of interacting proteins of Delta and Serrate in *Drosophila melanogaster* by STRING analyses. **(A)**: Interacting proteins of Delta. **(B)**: Interacting proteins of Serrate. Note: N, Notch; mib1, Mind-bomb; neur, Neuralized; Su(H), Suppressor of hairless protein; Kuz, Kuzbanian; Psn, Presenilin; Ser, Serrate; fng, Fringe; Dl, Delta; sca, Scabrous; H, Hairless; dx, Deltex; mam, Mastermind.

At present, no respective data of BPH is available from public databases; however, the function of the Notch signaling pathway is conserved in most species. Therefore, we chose *D. melanogaster* as the model insect to predict the partner proteins of Dl and Serrate (Ser, homologous to Jag). Through STRING analysis, both Dl and Ser were found to be closely related to seven proteins including Notch, Mind-bomb, Neuralized, Suppressor of hairless protein, Kuzbanian, Presenilin, and Fringe (Figure 10). Furthermore, Dl interacts specifically with Scabrous and Hairless (Figure 10A), while Ser interacts specifically with Deltex and Mastermind (Figure 10B).

## Discussion

Due to the high accuracy of RNAi treatment compounds, high degradability, and environmental friendliness, RNA biopesticides are considered “the third revolution in the history of agriculture” (Li et al., 2022; Yang et al., 2022), and RNAi biopesticide is a promising novel approach in the control of BPH. In the present study, two key ligand genes (*dl* and *jag*) of the Notch signaling pathway were selected and were systematically evaluated regarding their potential as targets for RNAi biopesticides. Short-winged BPHs are more reproductive and destructive, whereas long-winged adults are more migratory (Xu et al., 2015; Zhang et al., 2017). This difference in characteristics may require differentiated control measures. It is almost impossible to distinguish the wing morph of adults before emergence, which limits differentiated research on nymph control. To address this problem, we cultivated a stably long-winged (Macropterous strain) and a short-winged population (Brachypterous strain) of BPH strains through more than 70 generations of continuous cultivation, and we evaluated the RNAi effects of *Nldl* and *Nljag* in these two strains. In our observations and some previous reports, the RNAi experiment performance in these two strains was not significantly different from that of wild-type BPH (Li et al., 2019a; 2019b, 2021). These cultivated strains have thus become precious and scarce materials for the research on BPH.

We first cloned the cDNA sequences of *dl* and *jag* in both strains and found no nucleic acid sequence mutations, suggesting that there is no need to distinguish strains when designing dsRNA for interference. We selected 15 species for phylogenetic analysis of Dl and Jag respectively, however, the two proteins did not produce congruent results, suggesting evolutionary and functional differences between them. Although *dl* and *jag* have high homology with various insects (no more than 76%), we can still select suitable sequences to specifically interfere with BPH. If there are enough nucleic acid databases available in the future, we can even develop ds*dl* or ds*jag* to control multiple pests simultaneously. The related genes of the Notch signaling pathway mainly exist in animals, indicating that it should be difficult to interfere with the homologous genes in rice when ds*Nldl* and ds*Nljag* are applied as RNAi biopesticides. Furthermore, the identity of *dl* and *jag* with human homologous genes is far lower than those of insects. As long as the dsRNA targeting sequences are properly screened and designed, they will not be harmful to humans.

Through spatiotemporal expression analysis by qPCR, we found that *Nldl* and *Nljag* were higher expressed in the head and abdomen, indicating that these two genes were involved more in neural activity and metabolism. In particular, the expression level of *Nljag* was the highest in the thorax of fourth-instar nymphs of the Macropterous strain, representing the critical period of wing-pad development (Li et al., 2019b; Zhang et al., 2019). We therefore speculate that this gene may be involved in the wing development of BPH, as confirmed by adult wing deformity caused by *Nljag*-knockdown.

Through RNAi treatment, we found that injection of BPH nymphs of both Brachypterous and Macropterous strain with ds*Nldl* or ds*Nljag* produced the expected lethal effects. ds*Nldl* at only 10 ng/nymph caused failure to emerge in >80% of the nymphs. Even if some treated nymphs can survive to adulthood, wing development deformities caused by dsRNA treatment may also reduce their mobility, fitness, and reproductive success. dsRNA treatment showed excellent inhibitory effects on the expression of target genes on days 1 and 5, which suggested that RNAi works rapidly and its effects are persistent in BPH. We have also tried to increase the injection dosage to detect whether the interference efficiency can be improved, but the change is not obvious. However, this does not prevent us from obtaining the stable adult phenotypes. The phenotypes after dsRNA treatment can obtain 100% consistent phenotypes regardless of whether the dosages were increased or decreased. Our findings support the potential of these two genes as RNAi biopesticide targets for controlling BPH. It should be noted that with the same injection dosage of ds*Nldl*, mortality differed between the Brachypterous and Macropterous strains. Interference efficiency of dsRNA was higher in the Macropterous than in the Brachypterous strain. This may be related to the physiological tolerance of different strains of nymphs. Rice at the yellow-ripe stage (relatively low nutritional value) can stimulate the development of more long-winged BPH, whereas rice at the tillering-stage (relatively high nutritional value) induces development of more short-winged adults (Liu et al., 2020; Li et al., 2021). Therefore, in the future control and when ds*Nldl* is used as an RNAi biopesticide, the dosage should be adjusted according to the different growth periods of rice.

To further explore the functions of Dl and Jag in insects, the associated proteins of these two ligands were predicted using STRING software. However, the database did not include data on *N. lugens*, nor on Hemiptera. Referring to the phylogenetic analysis of Dl, *N. lugens* is closely related to *D. melanogaster*, we thus used *D. melanogaster* as a model insect to predict the respective partner protein of Dl and Jag/Ser. As expected, most of these co-expressed proteins were associated with signal transduction and functioning of the Notch signaling pathway, and most of the predicted associated proteins of Dl and Jag/Ser were identical. According to our findings, the lethal effect produced by ds*Nldl* injection was stronger than that of ds*Nljag* treatment, and there were also obvious differences in the phenotype after emergence. We propose that these differences may originate from differences in associated proteins. In detail, Dl is specifically associated with Scabrous and Hairless, which may be responsible for the higher lethality. The Deltex and Mastermind associated with Jag/Ser may be the cause of the curling of wings in adults. However, these speculations need further verification.

Our study preliminarily investigated the expression profiles and functions of *dl* and *jag* in BPH and confirmed their application potential. This potential was manifested by the outstanding lethal and teratogenic effects of dl and jag on the BPH. We further propose dosages regarding target genes of RNAi biopesticide to control BPH. RNAi biopesticides can be delivered in a variety of ways, mainly divided into resistant plant-based plant-incorporated protectants and non-plant-incorporated protectants. It should be emphasized that studies have demonstrated that transgenic plants expressing dsRNA have poor control effects on a variety of piercing-sucking insects, including the BPH, mainly because these insects are difficult to effectively consume sufficient dsRNA during the feeding process (Kim and Zhang, 2022). Delivery of dsRNA by feeding is also a common method in research and production, especially for Coleoptera and Lepidoptera insects. But for oligophagous BPH, the attraction of food attractants is significantly lower than that of host rice plants in the field. Fortunately, RNAi biopesticides can also follow the application methods of traditional chemical pesticide and are designed to exert a pest control role by the means of spray or root-irrigation, although several problems remain to be addressed before practical application, including risk assessment, cost control, and application methods. No fully commercialized non-PIP RNAi-based biopesticide products have been released to the market thus far (Kunte et al., 2020; Liu et al., 2020). However, RNAi biopesticides bear considerable application value and are expected to become an important means of integrated pest control.

## Data Availability

The original contributions presented in the study are included in the article/Supplementary Material, further inquiries can be directed to the corresponding author.
